# Genotype-Phenotype Correlation for Predicting Cochlear Implant Outcome: Current Challenges and Opportunities

**DOI:** 10.3389/fgene.2020.00678

**Published:** 2020-07-14

**Authors:** Adrien A. Eshraghi, Sai P. Polineni, Camron Davies, David Shahal, Jeenu Mittal, Zaid Al-Zaghal, Rahul Sinha, Urmi Jindal, Rahul Mittal

**Affiliations:** ^1^Department of Otolaryngology, Miller School of Medicine, University of Miami Hearing Research Laboratory, Miami, FL, United States; ^2^Department of Neurological Surgery, Miller School of Medicine, Miami, FL, United States; ^3^Department of Biomedical Engineering, University of Miami, Coral Gables, FL, United States

**Keywords:** age-related hearing loss, genetic etiology, hearing loss, genotye–phenotype correlation, cochlear implant

## Abstract

The use and utility of cochlear implantation has rapidly increased in recent years as technological advances in the field have expanded both the efficacy and eligible patient population for implantation. This review aims to serve as a general overview of the most common hearing disorders that have favorable auditory outcomes with cochlear implants (CI). Hearing loss in children caused by congenital cytomegalovirus infection, syndromic conditions including Pendred Syndrome, and non-syndromic genetic conditions such as hearing impairment associated with *GJB2* mutations have shown to be successfully managed by CI. Furthermore, cochlear implantation provides the auditory rehabilitation for the most common etiology of hearing loss in adults and age-related hearing loss (ARHL) or presbycusis. However, in some cases, cochlear implantation have been associated with some challenges. Regarding implantation in children, studies have shown that sometimes parents seem to have unrealistic expectations regarding the ability of CI to provide auditory rehabilitation and speech improvement. Given the evidence revealing the beneficial effects of early intervention via CI in individuals with hearing disorders especially hearing loss due to genetic etiology, early auditory and genetic screening efforts may yield better clinical outcomes. There is a need to better understand genotype-phenotype correlations and CI outcome, so that effective genetic counseling and successful treatment strategies can be developed at the appropriate time for hearing impaired individuals.

## Introduction

Hearing loss is a common neurosensory disorder affecting humans. Studies have long documented the significant prevalence, financial burden, and societal ramifications of hearing loss (Mathers et al., 2002; Dalton et al., 2003; Nelson et al., 2005; Agrawal et al., 2008; Vos et al., 2017; Graydon et al., 2019; Ronner et al., 2020). A World Health Organization (WHO) report on the global prevalence of hearing loss estimates that 466 million people around the world presently live with disabling hearing loss (WHO, 2018 Hearing Loss Estimates). In this population, seven percent, or 34 million, of the affected individuals are 14 years old or younger (WHO, 2018 Hearing Loss Estimates). While aging is the leading cause of hearing loss in adults, the causes and pathophysiology of hearing loss are numerous and vary among different individuals.

There are three main types of hearing loss: sensorineural, conductive, and mixed (Gifford et al., 2009; Cunningham and Tucci, 2017). Of these three types of hearing loss, sensorineural hearing loss (SNHL) is the most prevalent and can be the result of abnormal development (or function) of the hair cells (HCs) in the inner ear or due to abnormalities of the vestibulocochlear nerve (CN VIII) (Loss, 2012). SNHL can present either congenitally or in adulthood via a spectrum of conditions and diseases that will be discussed in this review. As SNHL tends to be a progressive condition characterized by permanent, irreversible damage to the inner ear hair cells or vestibulocochlear nerve, effective treatment modalities have yet to be developed (Rubel et al., 2013; Blanc et al., 2020; Farooq et al., 2020). Hearing aids in combination with rehabilitation programs can be used to manage the effects of SNHL in adults and children with mild to moderate SNHL (Walling and Dickson, 2012; Butler et al., 2013; Tomblin et al., 2014; Kim et al., 2020). However, hearing aids rely on the presence and proper function of inner ear hair cells. Hearing aids are not appropriate for individuals having severe or profound SNHL or individuals with poor discrimination, where the remaining number of inner ear hair cells are not sufficient for stimulation of the auditory cranial nerve. For these individuals, a cochlear implant (CI) is a viable option to provide auditory rehabilitation.

**FIGURE 1:**
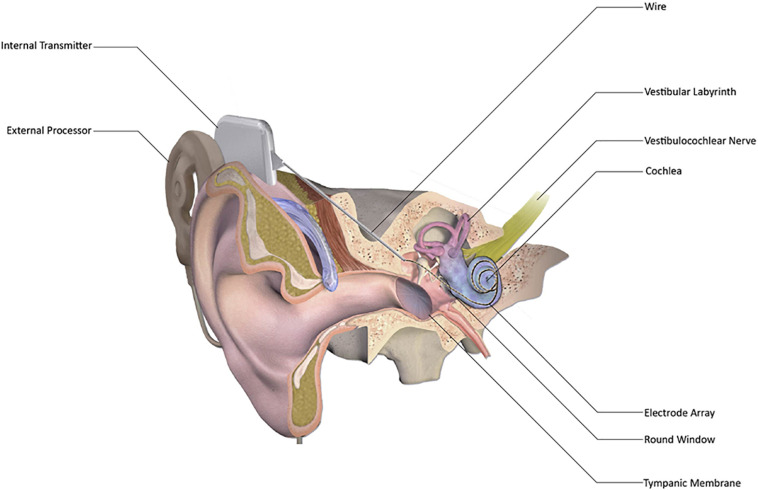
Schematic representation of cochlear implant (CI). CI has external processor that consists of a microphone, a digital sound processor, a battery, and an antenna that communicates with the internal processor. The internal processor receives sound information from the external processor and transmits it to the cochlea via a wire and electrode array inserted into the scala tympani, thereby enabling sound perception. [Adapted from “Cochlear Implant” by BruceBlaus {Blausen.com staff (2014). “Medical gallery of Blausen Medical 2014”. WikiJournal of Medicine 1 (2)} used under CC BY 3.0/Modified for clarity https://doi.org/10.15347/wjm/2014.010].

Despite the initial introduction in 1957 and commercialization, CI became widely used in the last 20 years to provide auditory rehabilitation to individuals having severe to profound SNHL (Macherey and Carlyon, 2014; Eshraghi et al., 2017; Sousa et al., 2018; Boisvert et al., 2020; Sharma et al., 2020). The most distinguishing feature between CI and hearing aids is that hearing aids merely amplify sounds so that they can be detected by the remaining inner ear hair cells in a patient with mild to moderate hearing loss. CI directly stimulates the auditory nerve, bypassing the functionality, or lack thereof, of the inner ear hair cells altogether (Eshraghi et al., 2012). Cochlear implants consist of five parts – an external microphone, an external speech processor, an external transmitter, an internal receiver, and an internal electrode array (Figure 1; Lenarz et al., 2012; Roche and Hansen, 2015). These work in concert to receive sounds from the external environment, convert them to radio waves that cross the skin from the transmitter to the receiver, further convert the radio waves to electrical impulses, and then send these electric impulses along the electrode array to stimulate the auditory nerve (Figure 1; Niparko and Marlowe, 2010; Roche and Hansen, 2015; Carlson, 2020).

Given that both children and adults who suffer from severe to profound SNHL are eligible for CI, 324,200 cochlear implants have been implanted worldwide as of December 2012, with 58,000 devices implanted in adults and 38,000 in children in the U.S. alone (NIH Publication No. 00-4798,2016). Note, however, that these numbers are dwarfed by the estimated 3.8 million Americans age 50 or older who wore a hearing aid at some time between 1999 and 2006 (Chien and Lin, 2012). However, given that CI remains the best rehabilitation option for both children and adults who suffer from severe to profound SNHL, an examination of the clinical conditions which cause SNHL that can be treated via CI may prove valuable. The clinical outcomes of CI may be influenced by the genetic etiology underlying hearing loss (Table 1). Additionally, an understanding of the current challenges associated with CI and recent technological advances in the field may help us in anticipating future directions for the clinical care of patients via CI.

**TABLE 1:** Genotype-phenotype correlation and cochlear implantation outcomes in most common causes of Non-Syndromic Deafness.

Gene	Protein – function	Area of expression	Benefits
*ACTG1*	γ−Actin – Microfilament, structural protein	Main actin isoform in the IHC and OHC in the cochlea	CI (EAS) was an effective therapeutic intervention for patients with *ACTG1* mutations (Miyagawa et al., 2015b; Usami et al., 2020).
CDH23*	CDH23 – Cell adhesion protein	IHC and OHC in the cochlea	CI (EAS) performed with good stimulation outcomes and preserved hearing (Usami et al., 2012). *Limitations*: Implantation is often done in young children where PTAs are not available. Performance evaluated with ABR but ABR has lower fidelity at low frequencies (Usami et al., 2012).
*CHD7*	CHD7 –Chromodomain-helicase-DNA-binding protein 7	Transcription factor in SGN	8 patients had CI, 5 had favorable outcomes. Larger cochleovestibular nerve diameter and absence of severe mental retardation indicated favorable outcomes, better than the type of *CHD7* mutation. Two patients did not benefit from CI and underwent auditory brainstem implantation (Song et al., 2011; Eppsteiner et al., 2012). Recent systematic review indicates that CI is beneficial for patients with CHARGE syndrome (Amin et al., 2019) CI is strongly recommended in favorable cases, auditory brainstem implantation may be an alternative for patients with CHARGE syndrome who fail to benefit from CI (Song et al., 2011).
*PJVK*	Pejvakin – Stereocilliary rootlet protein	HC, SGNs and brainstem auditory nuclei	3/7 patients with poor CI outcomes had homozygous *PJVK* mutations. These patients scored worse on CAP, SIR, and speech perception scores than match controls. *Limitations:* Small sample size and homogenous population limit generalizability (Wu C.-C. et al., 2015).
*GJB2*	Connexin 26 (Cx26) – gap junction protein	Spiral ligament, supporting cells, basal cells of the stria vascularis, and limbal fibrocytes (Kikuchi et al., 1995)	Open-Set speech perception improved in all *GJB2*-positive children (Cullen et al., 2004) Children with *GJB2* (*n* = 12, 9 with two mutated alleles) mutations also showed excellent CAP scores at 3 years after implantation (Wu et al., 2011) No differences in CI speech perception performance between the children with connexin mutations and children with deafness of unknown etiology (Taitelbaum-Swead et al., 2006) *Limitations*: Compared to children with non-*GJB2*-related severe hearing loss, *GJB2*-positive children did not show any statistically significant difference in improvement (Cullen et al., 2004; Karamert et al., 2011).
*LOXHD1*	LOXHD1 – Lipoxygenase Homology Domains 1	HC and in the membrane of stereocilia (Grillet et al., 2009)	All CI patients with available data indicated favorable outcomes, good candidates for CI/EAS (Maekawa et al., 2019) *Limitations:* Data only available for 4/8 patients with CI (Maekawa et al., 2019).
*MYO15A*	MYO15A – Motor protein	Critical in elongation of stereocilia and actin organization in HCs	Four children with bilateral CI indicate good results in speech discrimination scores (Miyagawa et al., 2015a) Good CI performance in *MYO15A* variants (Liu et al., 2019) *Limitations*: Most of the mutations appear unique to the Japanese population, this limits generalizability (Miyagawa et al., 2015a).
*MYO6*	Unconventional Myosin-6 *–* Motor Protein	Cuticular plate region of IHC and OHCs, role in stereocilia formation, and anchor (Miyagawa et al., 2016)	Good post-operative performance in Freiburger monosyllable word test and Oldenburger sentence test in background noise and in quiet. No vestibular dysfunctions or retrocochlear pathology in participating family members. Overall, CI outcomes in MYO6 are good (Volk et al., 2013; Miyagawa et al., 2015c; Liu et al., 2019). *Limitations*: 71 years old patient with Parkinson’s disease and dementia, had unsatisfactory CI outcome; 26% in monosyllable test at 70 dBSPL (Usami et al., 2020).
MYO7A*	MYO7A – Motor/Anchor protein	Stereocilia	Associated with improved results in Usher syndrome but age is most prognostic factor in CI performance with early CI achieving satisfactory auditory and speech outcomes (Xiong et al., 2019) Case study: CI significantly improved speech perception tests [monosyllable: 77%; word: 84%, sentence: 100% (Usami et al., 2020)].
*PCDH15*	Protocadherin-15 – structural protein	Tips of stereocilia, organ of corti, lateral wall, and SGN	4/7 patients with poor CI outcomes had bi-allelic PCDH15 mutations; outcomes were worse in CAP, SIR, and speech perception scores *Limitations:* Small sample size (*n* = 4) and homogenous population limit generalizability (Wu C.-C. et al., 2015).
*OTOF*	Otoferlin – presynaptic structure and Ca^2+^ ion sensor (Xiong et al., 2019)	IHCs and immature OHCs, required for synaptic exocytosis at ribbon synapses (Roux et al., 2006)	Important mutation leading to auditory neuropathy spectrum disorder (ANSD). 10 ANSD patients (age 1–5 years old) with *OTOF* mutations with stable hearing at, 89.0 ± 12.3 dBHL and absent ABR at 95 dBHL before surgery. Post implantation, all patients had excellent electrophysiological responses and auditory and speech performances. Excellent improvement in CAP and SIR scores, comparable to other genetic causes of SNHL after CI (Wu et al., 2018). Overall, good CI outcomes (Zhang et al., 2013; Chen et al., 2018; Wu et al., 2018) *Limitations:* Auditory improvement with age seen only in ∼20% of cases (Wu et al., 2018).
SLC26A4*	Pendrin – chloride−formate exchanger	Outer sulcus cells; regulates volume	CI before 3.5 years of age, patients show significantly higher CAP/SIR scores than those without mutations 3 years post-implant (Wu C.-M. et al., 2015) At 3 years after implantation, both homozygous and heterozygous children (*n* = 18 and *n* = 22 including heterozygotes) had better CAP scores than children with no detected mutation (*n* = 75) (Wu et al., 2011) *Limitations*: Effects on CI outcomes may depend on the age of implantation, children older than 3.5 years had no significant differences in post CI performance compared to those without mutations (Wu C.-M. et al., 2015).
*TMPRSS3*	TMPRSS3 – Type II Transmembrane Serine Protease	IHC, OHC, supporting cells, inner and outer sulcus cells, interdental cells, cochlear neurons, and SGN	Most CI cases show good outcomes. Two cases reported indicated poorer performances (Eppsteiner et al., 2012; Usami et al., 2020) Limitations: The causes of the discordant outcomes further investigation.

* Cause both syndromic and non-syndromic deafness CDH23, MYO7A, and MYO15A mutations were seen in both the pre- and post-lingual groups, indicating these genes may express variable phenotypes. HC: hair cell; IHC: inner hair cell; OHC: outer hair cell; SGN: spiral ganglion neuron; ANSD: auditory neuropathy spectrum disorder; EAS: electroacoustic stimulation; PTA: pure tone audiogram; CAP: categories of auditory performance test; SIR: speech intelligibility rating.

## Current Use of CI in Congenital Hearing Loss

One of the most significant effects of untreated SNHL in young children is delayed language, reading, and behavioral development (Blair et al., 1985; Nelson et al., 2020; Ronner et al., 2020; Williams et al., 2020). While children with mild SNHL may present with little to no developmental delays, children with severe hearing loss can benefit significantly from CI leading to early rehabilitation and training, even if they suffer from other disabilities (Smith et al., 2005; Wake et al., 2006; Edwards, 2007; Eshraghi et al., 2015; Mesallam et al., 2019; Guo et al., 2020; Williams et al., 2020). Thus, early identification of SNHL in children, and the disorders that cause this hearing loss and in which cochlear implantation is most effective can serve as a valuable treatment option for many children around the world.

SNHL in children can be congenital or acquired. Of children with congenital hearing loss, approximately 50% of cases are due to environmental causes, while another approximately 50% are due to genetic factors (Smith et al., 2005; Korver et al., 2017). Within the environmental causes of congenital hearing loss, the most common etiology is congenital cytomegalovirus infection, with congenital rubella syndrome, ototoxicity, prematurity, and asphyxiation also proving to be common causes (Barbi et al., 2003; Smith et al., 2005; Papsin and Gordon, 2007; Lanzieri et al., 2017; Angueyra et al., 2020). Studies have examined the efficacy of cochlear implantation in children afflicted by congenital cytomegalovirus (Dollard et al., 2007; Yamazaki et al., 2012; Hoey et al., 2017; Yoshida et al., 2017). After cochlear implantation, children deafened by congenital cytomegalovirus seem to show improved speech perception equivalent to non-cytomegalovirus congenitally deaf children, though speech production lagged when compared to the control group. It may be attributable to the other comorbidities related to congenital cytomegalovirus (Philips et al., 2014; Yoshida et al., 2017; Kraaijenga et al., 2018).

The many genetic causes of congenital hearing loss in children can be further categorized into syndromic and non-syndromic conditions, which, respectively, constitute 30% and 70% of the genetic causes of congenital SNHL (Smith et al., 2005; Korver et al., 2017). There are estimated several hundred syndromes suspected to involve hearing loss, some of the most common syndromic conditions include Alport syndrome, Pendred syndrome, Usher syndrome, and Waardenburg syndrome (Morton and Nance, 2006; Wémeau and Kopp, 2017; Gettelfinger and Dahl, 2018; Table 2). Some of the most common genes involved in hearing loss include mutations in collagen genes expressed in basement membranes such as *COL4A3*, *COL4A4*, and *COL4A5*.

**TABLE 2:** Most common syndromic causes of deafness.

Syndrome	Genes	Benefits	Limitations
Alport Syndrome	*COL4A3*, *COL4A4*, *COL4A5*	– Cause of post-lingual deafness – Cochlear implantation may prove to be a viable treatment option for the hearing loss characteristic of Alport syndrome (Bittencourt et al., 2012)	– Very limited published information concerning CI outcomes and Alport Syndrome
Jervell and Lange Neilsen	*KCNQ1* *KCNE1*	– Children under 2 years of old had an average score of 33 of 35 on the littlEARS test. Children over 2 years of old tested score averaged a score of 6 out of 10 on a proprietary speech perception test. All children were able to be mainstreamed into regular schooling (Vivero et al., 2010) – Literature review of nine studies indicates good auditory outcomes post CI (Eftekharian and Mahani, 2015)	– Long QT syndrome present in JLN presents a risk during CI surgery. It may be prudent to perform ECG in suspected patients. Reports of defibrillation needed after CI surgery (Kaneshiro et al., 2018)
Pendred	SLC26A4*	– 2.66-fold increase in phoneme scores post-operatively in children over a 48 months post-op time course (van Nierop et al., 2016) – 5.4-fold increase in phoneme scores in adults over a 12 months post-op time course (van Nierop et al., 2016) – Significant improvement in speech perception in children when compared to the reference group (*p* = 0.031) (van Nierop et al., 2016) – Statistically significant improvements in basic sound perception (*p* = 0.002), advanced sound perception (*p* = 0.004), speech production (*p* = 0.018), and activity limitations (*p* = 0.018) (van Nierop et al., 2016)	– Non-significant difference in adults with Pendred syndrome who underwent CI when compared to the adult reference group (*p* = 0.094) (van Nierop et al., 2016) – No significant improvement in speech perception in either children or adults when compared to patients with non-Pendred enlarged vestibular aqueduct who underwent CI (van Nierop et al., 2016) – No significant improvement in self-esteem (*p* = 0.164) or social interaction (*p* = 0.107) (van Nierop et al., 2016)
Usher Type I	MYO7A*, CDH23*, *USH1C* *PCDH15* *USH1G* *CIB2*	– Closed-Set Word Ability (speech perception): all patients aged 3 and older, except 1, improved from 0 to 100% perception. 2 patients under the age of 3 showed 50% perception at follow-up while the remaining 2 showed 100% (Loundon et al., 2003) – Open-Set Words (speech perception): All patients started at 0% perception pre-implant and, at post-implant follow-up, 4 still had 0%, 2 had 25%, 3 had 50%, 2 had 75%, and 2 had 90% perception scores (Loundon et al., 2003) – Speech production showed statistically significant improvement (*p* = 0.02) in those under 18 years of age. The adults all had complex sentence speech production prior to implantation (Loundon et al., 2003)	– CI was very effective in improving speech perception and production but results were unfortunately not uniformly successful (Loundon et al., 2003)
Waardenburg	*PAX3* *MITF* *EDNRB* *EDN3* *SOX10*	– 7 pediatric patients (mean age 37 ± 20 months) with a duration of CI use for 69 ± 42 months were retrospectively studied – Closed-Set Word Ability assessment via the Early Speech Perception test: 5 of the 7 patients scored 100%, 1 scored 79% (patient had a wound seroma), and 1 was not tested – Open-Set Word Ability via the Phonetically Based Kindergarten Test: 6 of the 7 were assessed and scores of 84, 80, 80, 60, 52, and 40% (patient had a wound seroma) were reported – No major complications were associated with cochlear implantation	– No control group against which to compare – Small, specific study population

*Cause both syndromic and non-syndromic deafness.

Alport syndrome can be inherited in either an X-linked or autosomal recessive fashion and leads to hearing loss, among other symptoms (Kruegel et al., 2013; Phelan and Rheault, 2018; Watson et al., 2020). The hearing loss in Alport presents as high tone hearing loss around the age that children enter primary school or later in the second decade of life (Harvey et al., 2001; Kruegel et al., 2013). This is an example of post-lingual deafness, i.e., deafness that occurs after the patient has already acquired speech and language. Cochlear implantation may prove to be an effective treatment option for the hearing loss due to Alport syndrome (Bittencourt et al., 2012).

Pendred syndrome, the most common syndromic cause of SNHL, is another syndromic condition that is inherited in an autosomal recessive fashion and usually causes congenital SNHL, often at birth, via enlarged vestibular aqueduct, a common inner ear deformity (Smith et al., 2005; Morton and Nance, 2006; Azaiez et al., 2007; Wémeau and Kopp, 2017). Studies of cochlear implantation in children with Pendred syndrome found that they have better speech perception compared to children who are deaf due to enlarged vestibular aqueduct not caused by Pendred syndrome despite the possibility of mild surgical complications due to the inner ear malformations caused by Pendred (Kontorinis et al., 2011; van Nierop et al., 2016; Mikkelsen et al., 2019; Mey et al., 2020). Other studies indicate that early diagnosis of SNHL, followed by cochlear implantation and rehabilitation, is also beneficial in Usher and Waardenburg Syndromes (Young et al., 1995; Loundon et al., 2003; Cullen et al., 2006; Hartel et al., 2017; Alzhrani et al., 2018; Nair et al., 2020).

There are several other genetic causes of congenital hearing loss, and the most common are outlined in Table 1 along with their CI outcomes. Interestingly, many of these mutations follow a relatively consistent pattern regarding good CI performance outcomes. It appears, however, that the genes expressed in or affecting the spiral ganglion are associated with poorer outcomes than those functioning elsewhere. This is mostly consistent with and expands upon the Spiral Ganglion Hypothesis (Eppsteiner et al., 2012). Recent studies have reinforced this finding suggesting that genetic mutations affecting the spiral ganglion neurons (SGNs) have significantly poorer outcomes in speech perception post-CI compared to mutations affecting the cochlea or inducing other forms of cochlear damage (Shearer et al., 2017). Additionally, it was found that performance outcomes were more variable if the mutation was neural in character as opposed to sensory (Shearer et al., 2017). This further speaks to the necessity of functioning neural circuits in capturing a CI’s output. Moreover, another interestingly consistent finding across studies was that currently unknown mutations had significantly worse outcomes than those currently known etiologies (Lee et al., 2020). This implies that those with known mutations are more amenable to cochlear implantation than those without currently identifiable variants. It also creates a call to action to investigate further and identify these unknown genetic etiologies of non-syndromic deafness to better serve these patient population.

In the following section, we will discuss the outcomes of CI in relation to the genetic etiology of hearing loss based on the expression of genes in the cochlea or SGNs.

### Intra-Cochlear Genes

*ACTG1* codes for γ−Actin, the main actin isoform found in IHC and OHC and is vital for cytoskeletal maintenance (Belyantseva et al., 2009). Mutations in this gene result in severe hearing loss with rapid progression within the first or second decade of life. This severe phenotype and rapid progression make an early diagnosis and intervention paramount in ensuring optimal CI performance. Fortunately, CI/electroacoustic stimulation (EAS) (cochlear implant that uses both electrical and acoustic stimulation) has shown to be effective therapeutic interventions in patients with *ACTG1* mutations (Miyagawa et al., 2015c; Usami et al., 2020). One study indicated poor CI outcomes for a small group of Norwegian adults, however, this patient population was not considered representative of the average population as they were in late adulthood, retained the use of sign language as their primary communication, and did not attend audiological speech rehabilitation (Rendtorff et al., 2006; Park et al., 2013). Overall, current evidence supports early CI/EAS in patients with *ACTG1* mutations (Lee et al., 2018).

*CDH23* codes for cadherin 23 found in the HCs of the cochlea. CDH23 interacts with protocadherin 15 and is vital in stereocilia formation, specifically, tip-link filaments (Kazmierczak et al., 2007). Phenotypically, frameshift mutations in CDH23 cause Usher syndrome 1D, and missense mutations cause non-syndromic hearing loss, specifically DFNB12 (Usami et al., 2020). Importantly, serial audiograms of patients with *CDH23* mutations are characterized by progressive hearing loss with some residual hearing (Miyagawa et al., 2012). The progressive hearing loss combined with residual hearing makes patients with *CDH23* mutations good candidates for CI/EAS. Indeed, studies have shown that EAS results in good stimulation outcomes with preserved hearing (Usami et al., 2012). However, it is essential to note that these performance outcomes are limited in that pure-tone audiograms (PTAs) are not available in young children and auditory brainstem responses (ABRs) have lower fidelity at low frequencies limiting interpretation (Usami et al., 2012).

*LOXHD1* codes for a lipoxygenase domain that localizes to hair cell membranes and is vital for their function. Patients with *LOXHD1* mutations have early onset high-frequency hearing loss that progresses at different rates, culminating in a severe phenotype (Maekawa et al., 2019; Usami et al., 2020). The mutations in this gene also display favorable CI outcomes. A recent Japanese study demonstrated that four individuals with *LOXHD1* mutations scored over 90% correct on monosyllable, word, and sentence perception tests 6 months post-CI (Maekawa et al., 2019). However, data was only available for a small patient population. Despite this limitation, patients with *LOXHD1* remain good candidates for CI/EAS.

*PCDH15* codes for protocadherin 15, the counterpart to cadherin 23 and is expressed in the tips of stereocilia. Phenotypically, a mutation in *PCDH15* is associated with both non-syndromic hearing loss or Usher syndrome type 1. Interestingly, unlike mutations in *CDH23*, *PCDH15* mutations are associated with poor CI outcomes. In one study, 4/7 patients with poor CI outcomes were found to have bi-allelic *PCDH15* mutations; performance was worse in the categories of auditory performance test (CAP), the speech intelligibility rating (SIR), and speech perception scores compared to matched controls. This study was limited by its small sample size and homogenous, Han Chinese population (Wu C.-C. et al., 2015). More recent studies have shown that *PCDH15* is highly expressed outside of the cochlea with the highest amounts in the SGN (Nishio et al., 2017). Damage to structures outside of the cochlea, especially the SGN, is likely the cause of poor outcomes in these patients.

*MYO7A* codes for motor and anchor proteins found in stereocilia in the cochlea. Mutations in *MYO7A* cause a wide range of phenotypes, including Usher Syndrome 1B and various forms of non-syndromic hearing loss (Usami et al., 2020). Thus far, previous studies examining *MYO7A* mutations have shown positive outcomes after CI, especially in Usher syndrome. One recent case study observed significantly improved speech perception scores (monosyllable: 77%; word: 84%, sentence: 100%) after CI (Usami et al., 2020). The most important prognostic factor appears to be the age of CI, with younger patients associated with better outcomes in auditory and speech performance (Xiong et al., 2019).

*MYO15A* codes for a similar motor protein that is essential for the regulation of stereocilia elongation and organization in the cochlea (Usami et al., 2020). Aberrant formation of stereocilia due to mutations in these motor proteins impair noise transduction to the SGN. *MYO15A* mutations are known to cause both severe pre-lingual hearing loss or milder post-lingual, progressive hearing loss phenotype (Miyagawa et al., 2015b). Overall, studies have reported good performance outcomes post-CI (Miyagawa et al., 2015b; Liu et al., 2019). However, most of these studies were conducted in homogenous Japanese population, limiting their generalizability.

*MYO6* codes for an unconventional myosin protein; unconventional in that it is a reverse-direction motor protein that moves toward the negative end of actin filaments and functions mainly in intracellular transport where it helps maintain the structural integrity of hair cells. Phenotypically, bi-allelic mutations lead to profound autosomal recessive hearing loss, while heterozygous missense mutations cause autosomal dominant hearing loss, albeit with a later onset and a milder phenotype. Post-CI performance indicates good outcomes in a variety of tests, including the Freiburger monosyllable word test and Oldenburger sentence test (Volk et al., 2013; Miyagawa et al., 2015a; Liu et al., 2019). Due to the limited number of cases reported, any adverse outcomes are also worth noting; one such case was a 71 years old patient with Parkinson’s disease and dementia that scored only 26% in monosyllable test at 70 dBSPL post-implant (Usami et al., 2020).

*OTOF* codes for otoferlin, a presynaptic calcium ion sensor expressed in IHCs and immature OHCs, which is required for synaptic vesicle-plasma membrane fusion (Roux et al., 2006). Mutations in *OTOF* are an important cause of auditory neuropathy spectrum disorder (ANSD), a hearing disorder characterized by a functional cochlea but a non-functional auditory nerve. So while sound transduction may occur, the signals are not sufficiently transmitted to the brain (Roush et al., 2011). Phenotypically, *OTOF* mutations lead to severe to profound non−syndromic pre-lingual hearing loss (Iwasa et al., 2019). Patients with *OTOF* mutations post-CI have relatively good outcomes (Zhang et al., 2013; Chen et al., 2018). One study of 10 ANSD patients (age 1–5 years old) with *OTOF* mutations with stable hearing before surgery showed excellent electrophysiological responses, auditory and speech performances, as well as improvement in CAP and SIR scores, comparable to other genetic causes of SNHL after CI (Wu et al., 2018). Good CI performance is likely seen in *OTOF* mutations as the SGN and auditory nerves are preserved, only the coupling between the cochlea and the nerves is disrupted. Interestingly, improvement with age is seen in approximately 20% of ANSD cases (Wu et al., 2018). This makes an accurate genetic test paramount in these patients.

### SGN

*CHD7* codes for a DNA binding protein expressed throughout the body, including the inner ear, that is thought to play a role in the organization of chromatin. *CHD7* mutations are the most common cause of CHARGE syndrome, a constellation of developmental abnormalities which include coloboma, heart defects, atresia choanae, growth retardation, genital abnormalities, and ear abnormalities. Regarding hearing loss specifically, nearly all patients have ear malformations, which lead to a mixed conductive and sensory neural hearing loss. About 40% of CHARGE patients have profound hearing loss, while 80% have a less severe hearing impairment (Amin et al., 2019). Overall, there seems to be an improvement in audiological outcomes post CI, however, they were inferior to outcomes of patients without CHARGE syndrome. The consensus is that CI is strongly recommended in favorable cases, i.e., those with large cochleovestibular nerve diameter and absence of severe mental retardation (Lanson et al., 2007; Song et al., 2011; Ricci et al., 2014; Amin et al., 2019). In unsuccessful CI cases, auditory brain stem implantation was suggested as an alternative (Song et al., 2011; Eppsteiner et al., 2012). However, the limited benefits of this more complex surgery must be weighed against the possibility of complications and the fact that usage of brain MRI might be limited for these patients in the future.

*PJVK* (previously *DFNB59*) codes for Pejvakin, a stereociliary rootlet protein expressed in HCs, SGN, and auditory brain stem nuclei (Usami et al., 2020). Phenotypically, patients with *PJVK* mutations have shown variable expressivity with some exhibiting ANSD, while others have hearing loss of cochlear origin (Kazmierczak et al., 2017). Unlike many of the previously mentioned mutations *PJVK*, is associated with poor CI performance. In a recent study, 3/7 patients with poor CI outcomes were found to have homozygous *PJVK* mutations. These patients scored worse on CAP, SIR, and speech perception scores than matched controls (Wu C.-C. et al., 2015). Given this small sample size and homogenous population, these conclusions have limited generalizability and need to be confirmed in future investigations. It is likely that the wide distribution of Pejvakin in the SGN and auditory brain stem nuclei impair CI signal propagation.

### Other

Mutations in genes *GJB2*, which codes for Gap Junction Beta-2 protein, also known as connexin 26 (Cx26), are responsible for approximately half of the cases of hereditary SNHL in developed nations (Smith et al., 2005; Chan and Chang, 2014). Cx26 is associated with hair cells in the cochlea but not the auditory nerve function; consequently, *GJB2* mutations preserve a potential pathway for auditory rehabilitation via cochlear implantation. Phenotypically, *GJB2* mutations are variable with frameshift mutations result in more severe phenotypes compared to missense mutations (Tsukada et al., 2010). Post-CI performance is overall positive (Cullen et al., 2004; Karamert et al., 2011; Yan et al., 2013). In another study, children with *GJB2* (*n* = 12, including nine with two mutated alleles) mutations had excellent CAP scores 3 years after implantation (Wu et al., 2011).

*SLC26A4* codes for pendrin, a chloride-formate exchanger expressed in outer sulcus cells that regulates volume. *SLC26A4* mutations are associated with a large range of phenotypes from Pendred Syndrome to varying degrees of non-syndromic hearing loss, the latter of which we will discuss here. Hearing loss is sometimes progressive but is often confined to the higher frequencies with preserved hearing at lower frequencies. This makes patients with *SLC26A4* mutations good candidates for CI/EAS. Indeed, studies have shown that post-CI patients have significantly higher CAP/SIR scores than those without mutations 3 years post-implant (Wu C.-M. et al., 2015). Moreover, at 3 years after implantation, both homozygous and heterozygous children with mutated *SLC26A4* alleles (*n* = 18 and *n* = 22 including heterozygotes) demonstrated a better CAP score than children with no detected mutation (*n* = 75) (Wu et al., 2011). Age is an essential factor in CI performance with this mutation, children older than 3.5 years had no significant differences in post CI performance compared to those without mutations (Wu C.-M. et al., 2015).

*TMPRSS3* codes for a type II transmembrane serine protease expressed in hair cells, supporting cells, sulcus cells, interdental cells, cochlear neurons, and the SGN. *TMPRSS3* mutations can lead to either a severe, pre-lingual hearing loss phenotype or a milder, later−onset, progressive, post-lingual hearing loss. In both cases, higher frequencies are typically lost first (Usami et al., 2020). However, the current evidence on post-CI performance outcomes is not clear. One recent study with five subjects has indicated that post-implantation performance results were far below average, which correlates with the expression of *TMPRSS3* in the human spiral ganglia (Tropitzsch et al., 2018). This was supported by an older study demonstrating that two cases showing poor performances both had *TMPRSS3* mutations (Eppsteiner et al., 2012). Conversely, excellent performance has also been seen in a number of studies (Elbracht et al., 2007; Weegerink et al., 2011; Miyagawa et al., 2013). The cause of the discordant outcomes requires further investigation.

## CI and Acquired Hearing Loss

As opposed to congenital hearing loss, acquired hearing loss results from a non-genetic, exogenous, or idiopathic etiology. The most common acquired cause of SNHL in adults is presbycusis, the loss of hearing that accompanies the aging process (Keithley, 2019; Fischer et al., 2020; Wang and Puel, 2020). Other acquired causes of hearing loss in adults include noise exposure, ototoxic medications, and vascular changes resulting from chronic conditions (Cunningham and Tucci, 2017; Liberman and Kujawa, 2017). The most common idiopathic SNHL in adulthood is sudden SNHL (Kuhn et al., 2011; Leung et al., 2016). Cochlear implants are a safe and effective treatment option in many of these populations as well.

Age-related hearing loss (ARHL), or presbycusis, is a multifactorial disease with varying etiologies and can be attributed to progressive and irreversible degeneration of the cochlea, inner ear, or the auditory nerve (Yamasoba et al., 2013; Fischer et al., 2020; Wang and Puel, 2020). Given its progressive nature, ARHL affects older adults, and many studies have been conducted to assess the safety and efficacy of CI in this population. Several studies have shown improvements in audiometric outcomes and subsequent improvements in quality of life in patients 50 years of age or older, even when compared to younger adult populations, after cochlear implantation (Rafferty et al., 2013; Sanchez-Cuadrado et al., 2013). Furthermore, within an age range from 50 to over 70 years, there does not seem to be a statistically significant difference in benefits from cochlear implants and hearing aids performance in speech tests (Lenarz et al., 2012; Rafferty et al., 2013; Sanchez-Cuadrado et al., 2013). In our experience, CI is effective and safe in patients more than 80 years old (Eshraghi et al., 2015). The benefits seen in the adult population aged over 50 were comparable to those seen in adult populations aged 18–49, although older patients had slightly decreased hearing performance in noisy environments and detecting of monosyllabic words compared to their younger counterparts (Lenarz et al., 2013; Lundin et al., 2013). Generally, the evidence seems to support the notion that in the treatment of ARHL, CI performance is not constrained by age, especially when combined with appropriate auditory rehabilitation and counseling, and when no significant cognitive decline is present (Parham et al., 2013).

While age does not seem to impact the efficacy of CI in ARHL, an individual’s tendency towards developing ARHL appears partially due to certain polymorphisms of specific genes (Liu and Yan, 2007; Sliwinska-Kowalska and Pawelczyk, 2013). Indeed between 35% and 55% of ARHL may be heritable. In one of the largest and most extensive studies identifying loci for ARHL genes to date, researchers identified 44 independent loci associated with self-reported hearing difficulty or hearing aid use in those between 40 and 69 years old. Thirty-four of these loci were novel associations with hearing loss of any form (Wells et al., 2019). Surprisingly, the study only identified one of the ten currently known hearing loci associated with ARHL. Mainly these genes segregated around functions related to inner ear morphology, synaptic activities, nervous system processes, and cognition. Of the 44 total identified, four stood out as the most significant: *EYA4, NID2, ARHGEF28*, and *CTBP2.* A few genes have also been associated with other forms of hearing loss such as *CDH23*, which is associated with early onset congenital deafness (Wells et al., 2019). These findings suggest that many of the hearing loss related genes are variably expressed and previously known mild or severe mutations may have additional yet unrecognized phenotypes.

Additional studies have found other associations. Of note is *GSTT1*, which codes Glutathione S-transferase Theta-1, an enzyme that conjugates metabolites to reduce oxidative stress (Liu and Yan, 2007). The null genotype of *GSTT1*, which is found in 25–40% of the Caucasian population, increases an individual’s susceptibility to oxidative stress-induced ARHL (Rabinowitz et al., 2002; Sliwinska-Kowalska and Pawelczyk, 2013). Individuals with the non-functional *GSTT1* have a 3-fold increased risk of developing ARHL compared to the wild type (Bared et al., 2010; Angeli et al., 2012; Manche et al., 2016). While the presence of the functional, wild type genotype of *GSTT1* has not been shown to impart a protective effect toward SNHL, other genes do. Functional, wild type allele of *GSTM1*, a separate gene in the glutathione pathway present in up to 50% of the Caucasian population, appears to provide a protective effect against ARHL (Rabinowitz et al., 2002; Liu and Yan, 2007; Sliwinska-Kowalska and Pawelczyk, 2013).

Cochlear damage secondary to therapeutic medications, particularly aminoglycoside and cisplatin-induced ototoxicity, has been well documented (Selimoglu, 2007; Xie et al., 2011). Therapeutically, aminoglycosides inhibit bacterial initiation complexes by irreversibly binding to the 30S subunit of ribosomes. Conversely, cisplatin targets rapidly proliferating cancer cells, intercalating with their DNA, crosslinking DNA strands, interfering with DNA repair mechanisms, and causing DNA damage via ROS generation. Despite their dissimilar therapeutic mechanisms, with both drugs, ototoxicity is the by-product of inadvertent free radical generation in the inner ear, ultimately leading to SNHL (Karasawa and Steyger, 2015; Kirkim et al., 2015). Aminoglycoside-induced ototoxicity is characterized by the initial degeneration of outer hair cells (OHCs), followed by the loss of inner hair cells (IHCs) in cases of severe ototoxicity (Xie et al., 2011). This is generally accompanied by subsequent degeneration of nerve fibers and supporting cells (Selimoglu, 2007; Xie et al., 2011). Given the mechanism of action of these ototoxic agents, cochlear implantation could serve as an effective option to address SNHL caused by these medications.

The extent and success of auditory rehabilitation provided by CI depend on the type of ototoxic agent and underlying pathology of SNHL (Nichani et al., 2013). In the literature, there is an interesting case report of a 14 years old male patient who had received cochlear implantation at 35 months of age and demonstrated substantial improvement in speech awareness post-implant (Harris et al., 2011). The individual presented with localized fibroblastic osteosarcoma that was subsequently treated with cisplatin. The patient began to present with hearing difficulties following chemotherapy. Given that the patient’s childhood SNHL was of idiopathic origin, which suggests of already reduced hair cell function, it is possible that cisplatin could have exacerbated the patient’s existing damage to the auditory system. This case serves as a reminder to continually assess for and take into consideration the potential ototoxic effects of otherwise therapeutic medication, especially in those individuals who have preexisting mild, moderate, or even severe hearing loss.

Though rare in children, sudden sensorineural hearing loss (SSNHL) is a rapid onset deafness, usually of one ear, that affects 5–20 out of every 100,000 people (Stachler et al., 2012). Though the vast majority of cases are idiopathic, SSNHL is considered a medical emergency since early treatment with corticosteroids may be beneficial (Stachler et al., 2012). Over half of the people who experience SSNHL will undergo spontaneous partial or complete hearing recovery; nevertheless, bilateral symptoms, profound hearing loss, advanced age, and delays in treatment are negative prognostic factors (Sara et al., 2014; Edizer et al., 2015). If recovery is not achieved within 3 months after the onset of SSNHL, studies support that cochlear implantation may be beneficial in regaining hearing and reduction of SSNHL-associated tinnitus (Ramos et al., 2012; Blasco and Redleaf, 2014; Jo et al., 2015; Dagna et al., 2016).

## Challenges Associated With Cochlear Implantation

With cochlear implantation, as with any surgical procedure, there exists the potential for surgical complications and associated consequences. Despite advances in surgical techniques and the design of less traumatic electrodes, CI may be associated with the loss of residual hearing in the implanted ear (Boggess et al., 1989; Eshraghi et al., 2017). Research is still being conducted to better understand the molecular mechanisms that are associated with the loss of residual hearing following inner ear trauma due to the insertion of the cochlear implant electrode array. Our studies have demonstrated that electrode insertion leads to the activation of apoptotic and inflammatory pathways, which cause sensory cell damage in the cochlea and lead to the loss of residual hearing (Eshraghi et al., 2015). The apoptosis initially occurs in the support cells after electrode insertion trauma followed by apoptosis in the hair cells (Eshraghi et al., 2015). The degree of residual hearing loss varies in implanted individuals, and genetic factors may influence the activation of apoptotic and inflammatory pathways. This increased genetic predisposition to loss of residual hearing needs to be explored in further studies. A better understanding of genetic etiology implicated in the loss of residual hearing will help in developing novel treatment modalities.

Additionally, the individuals having auditory neuropathy spectrum disorder (ANSD) sometimes do not significantly benefit from CI depending on the site of lesion (Shearer and Hansen, 2019). In ANSD, the inner ear can detect sound but is unable to send signals to the brain (Norrix and Velenovsky, 2014; Kaga, 2016). The individuals with ANSD show abnormal auditory brainstem recordings but have preserved distorted otoacoustic emissions suggesting normal, functional auditory outer hair cells (De Siati et al., 2020). The site of lesion in ANSD may involve the inner ear, IHCs, synapses, SGNs, or the auditory nerve. Since CI outcome is speculated to depend on the functional SGNs, in ANSD patients where SGNs are affected, or there is degeneration of the auditory nerve, CI may not show adequate performance (Shearer and Hansen, 2019).

At a cursory glance, the importance of maintaining residual hearing is not immediately apparent as cochlear implants are placed in individuals who have severe to profound hearing loss, However, as we will discuss, studies have documented numerous benefits that maintenance of residual hearing after CI contributes positively to auditory rehabilitation and patient self-confidence. Furthermore, the preservation of residual hearing will continue to gain relevance as an important outcome of CI surgery as the eligibility criteria for implantation continue to broaden, and as patients who may have unilateral or moderate hearing loss become eligible to receive CIs. The reasoning for the importance of preserving residual hearing stems from the fact that it can augment both the frequency and range of hearing, especially at lower frequencies (Khater and El-Anwar, 2017). This augmentation may subsequently lead to better auditory rehabilitation and hearing performance (James et al., 2005; Chiossi and Hyppolito, 2017; Neben et al., 2018).

Increasing understanding of the importance of residual hearing, in concert with advances in CI technology, has led to the recognition of electrode array design and surgical techniques as the keystone principles that determine success in the preservation of residual hearing. A systematic review further exposited on these principles by identifying five factors that seem to determine hearing preservation during CI: (1) minimally invasive surgery, (2) suitable electrode insertion route, (3) gentle insertion technique, (4) control of inflammatory reaction after CI, and (5) atraumatic electrodes (Khater and El-Anwar, 2017). In summary, to ensure the greatest preservation of residual hearing, round window insertion seems to be preferred over the cochleostomy approach, and flexible electrode arrays are preferred over more static arrays (Khater and El-Anwar, 2017; Gautschi-Mills et al., 2019).

While preservation of residual hearing is currently a primary challenge associated with cochlear implantation and a topic of much research, there are other potential complications of CI surgery. Minor complications are mostly temporary and include post-operative dizziness, taste disturbances, transient facial nerve palsy, and the possibility of new-onset of tinnitus. The major complications include facial nerve stimulation, skin flap complications, and rarely infection (Kubo et al., 2005). Infection via bacterial meningitis after CI has been associated mainly with the use of implants that included a positioner – a wedge inserted next to the electrode that pushes the electrode against the cochlea to facilitate the transmission of the electrical signal (Reefhuis et al., 2003; Farinetti et al., 2014). However, these implants have now been removed from the market. Individuals at risk of major infection complication post-cochlear implantation include children with a history of inner ear malformation. These individuals can be identified before surgery by imaging technology or genetic testing.

## Patient Satisfaction

Improved post-operative performance on hearing loss has been well-received, with regards to patient satisfaction, in both adolescent and adult patient populations, with a few specific exceptions. A study used the Satisfaction with Amplification in Daily Life (SADL) questionnaire to evaluate patient satisfaction in children in Brazil who received CI as a means to treat auditory neuropathy-induced hearing loss. It was observed that overall satisfaction had a mean of 8, with a minimum of 7 and a maximum of 9, on a 10-point Likert scale (de Carvalho et al., 2015). The same study showed that subcategorizing satisfaction by positive effects, negative effects, services and costs, and personal image yielded similar optimistic results in all categories except personal image, where little improvement was seen in the adolescents’ perception of their social image (de Carvalho et al., 2015). Another study using the SADL questionnaire in a Brazilian cohort of adults with post-lingual hearing loss, found similar results as overall satisfaction with cochlear implantation belied a dissatisfaction with Personal Image and Costs and Services in 13.7 and 27.5% of the population, respectively (Buarque et al., 2014). Approaching patient satisfaction via other tools, particularly the International Classification of Functioning, Disability and Health, Child and Youth version (ICF-CY) and two study-specific questionnaires, a different group of researchers, compared Swedish children with cochlear implants to those with hearing aids. The study found that approximately half the children in both groups – 50% for CI and 41% for hearing aids – had hearing problems in large groups (Anmyr et al., 2011). However, children with hearing aids reported higher rates of hearing difficulties when participating in team sports or outdoor activities. When compared to utilization of CIs, hearing aid users showed a significantly lower pattern of using their hearing device citing forgetfulness, hearing aid functional difficulties, and considering the hearing aid to be “boring or ugly” (Anmyr et al., 2011).

Parents of Dutch adolescents with unilateral CI completed prospective surveys prior to surgery for a second implant and 12 and 24 months after the procedure (Sparreboom et al., 2012). This study found that parents’ preoperative expectations of performance far outpaced observed results 12 months after surgery, though self-reported positive changes were still observed and reported by a majority of the parents. Furthermore, 24 months after surgery, the preoperative expectations and post-operative results were found to be statistically comparable, suggesting that parents may not be fully aware of the time scale for positive hearing gains after implantation. A study that attempted to assess this same issue via a quality of life questionnaire found no significant differences in the quality of life of bilaterally deaf children with a CI and contralateral hearing aid versus bilaterally deaf children with bilateral hearing aids and normal-hearing children (Perez-Mora et al., 2012). Studies investigating long-term patient satisfaction with CI through young adulthood and beyond found similar results as many patients reported that the benefits of CI outweighed any potential loss of residual hearing or other negative side effects (Galvin et al., 2014; Erixon and Rask-Andersen, 2015).

## Conclusion and Future Directions

CI is widely used to provide auditory rehabilitation for a variety of hearing disorders in both children and adult populations. CI seems to be highly effective in the majority of cases of hearing loss. As CI technology becomes more refined, the patient population eligible for such implants will likely increase in the future. Furthermore, it is expected that genetic screening will continue to play a significant role for the identification of appropriate children and adults with congenital or inherited hearing disorders. The promising results of early intervention via CI as well as guidelines that formalize appropriate and timely screening and intervention could lead to population-level health improvements. However, some patients with hearing loss due to mutations in specific, genes especially those expressed in SGNs, do not benefit adequately from CI. Our understanding is still very limited regarding phenotype-genotype correlation and CI outcome, especially in minority and Hispanic populations leading to a significant knowledge gap. Given the high surgical implantation and clinical management cost of CI (>$1 million lifetime cost), prospective identification of the worst performers would reduce unnecessary procedures and healthcare costs as well as surgical risks. Since the severity of hearing loss due to genetic etiology varies in different ethnic groups and hence CI outcome, there is a need to establish genotype-phenotype correlations. Establishing a genotype-phenotype association will pave the way to expand the indication of CI to hearing-impaired individuals as determined by their genetic cause of HL and will provide genetic-based CI performance metrics. Better knowledge about genotype-phenotype correlation and CI outcome will help in providing effective auditory rehabilitation to hearing-impaired individuals in pursuit of improving their quality of life.

## Author Contributions

All authors listed have made a substantial, direct and intellectual contribution to the work, and approved it for publication.

## Conflict of Interest

AE is a consultant and received research funding from MED-EL Corporation. The remaining authors declare that the research was conducted in the absence of any commercial or financial relationships that could be construed as a potential conflict of interest.
